# SAPrIm, a semi-automated protocol for mid-throughput immunopeptidomics

**DOI:** 10.3389/fimmu.2023.1107576

**Published:** 2023-06-02

**Authors:** Terry C. C. Lim Kam Sian, Gabriel Goncalves, Joel R. Steele, Tima Shamekhi, Liesl Bramberger, Dongbin Jin, Mohammad Shahbazy, Anthony W. Purcell, Sri Ramarathinam, Stoyan Stoychev, Pouya Faridi

**Affiliations:** ^1^ Department of Medicine, School of Clinical Sciences, Faculty of Medicine, Nursing & Health Sciences, Monash University, Clayton, VIC, Australia; ^2^ Monash Proteomics and Metabolomics Platform, Department of Biochemistry and Molecular Biology, Monash Biomedicine Discovery Institute, Monash University, Clayton, VIC, Australia; ^3^ Department of Biochemistry and Molecular Biology, Monash Biomedicine Discovery Institute, Monash University, Clayton, VIC, Australia; ^4^ ReSyn Biosciences (Pty) Ltd., Pretoria, South Africa

**Keywords:** immunopeptidomics, human leukocyte antigen, DIA, cancer antigen, HLA-bound peptides

## Abstract

Human leukocyte antigen (HLA) molecules play a crucial role in directing adaptive immune responses based on the nature of their peptide ligands, collectively coined the immunopeptidome. As such, the study of HLA molecules has been of major interest in the development of cancer immunotherapies such as vaccines and T-cell therapies. Hence, a comprehensive understanding and profiling of the immunopeptidome is required to foster the growth of these personalised solutions. We herein describe SAPrIm, an Immunopeptidomics tool for the Mid-Throughput era. This is a semi-automated workflow involving the KingFisher platform to isolate immunopeptidomes using anti-HLA antibodies coupled to a hyper-porous magnetic protein A microbead, a variable window data independent acquisition (DIA) method and the ability to run up to 12 samples in parallel. Using this workflow, we were able to concordantly identify and quantify ~400 - 13000 unique peptides from 5e5 - 5e7 cells, respectively. Overall, we propose that the application of this workflow will be crucial for the future of immunopeptidome profiling, especially for mid-size cohorts and comparative immunopeptidomics studies.

## Graphical Abstract

A schematic overview of the workflow for SAPrIm. 1) Showing the sample lysis for extraction of HLA-peptide complexes by homogenisation using bead milling. 2) Leveraging the Kingfisher Duo capabilities for automated and mid-throughput HLA immunoaffinity purification. 3) Acid eluted peptides clean-up by removal of heavy chains contaminant derived HLA molecules and antibodies using C18 resin in a 96 well plate. 4) Analysis of the HLA peptides by data independent acquisition (DIA) with variable m/z windows to increase detection of HLA peptides.

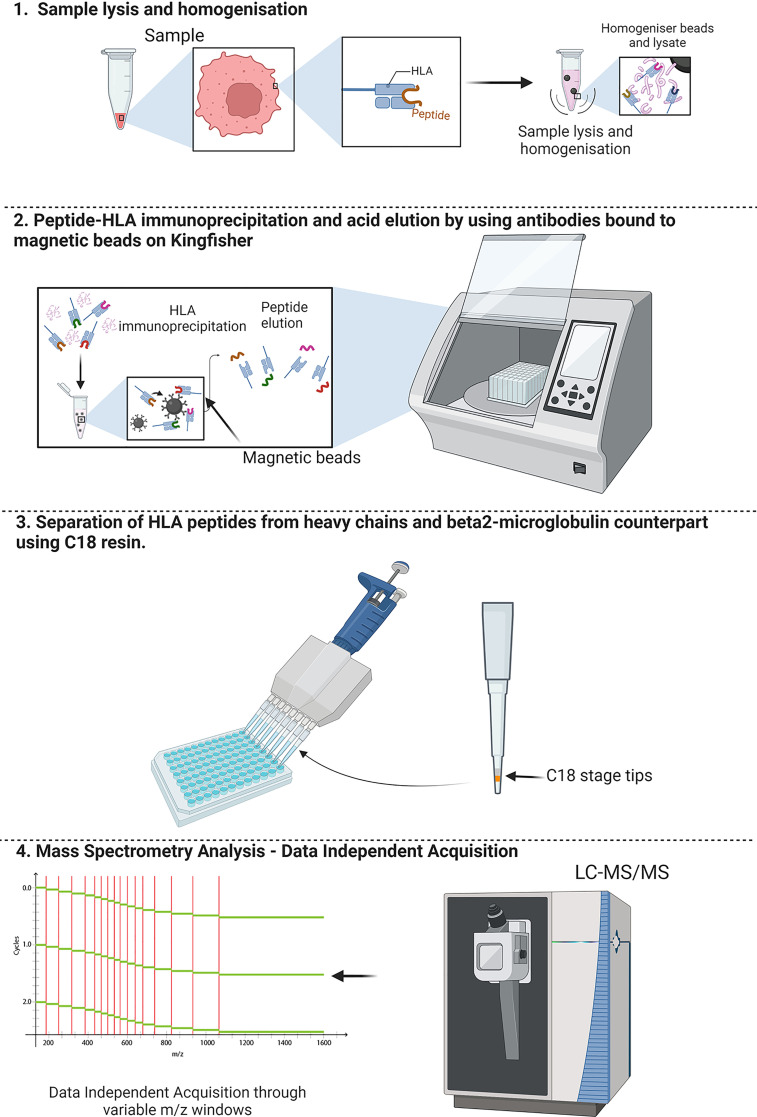

## 1 Introduction

**Figure 1:**
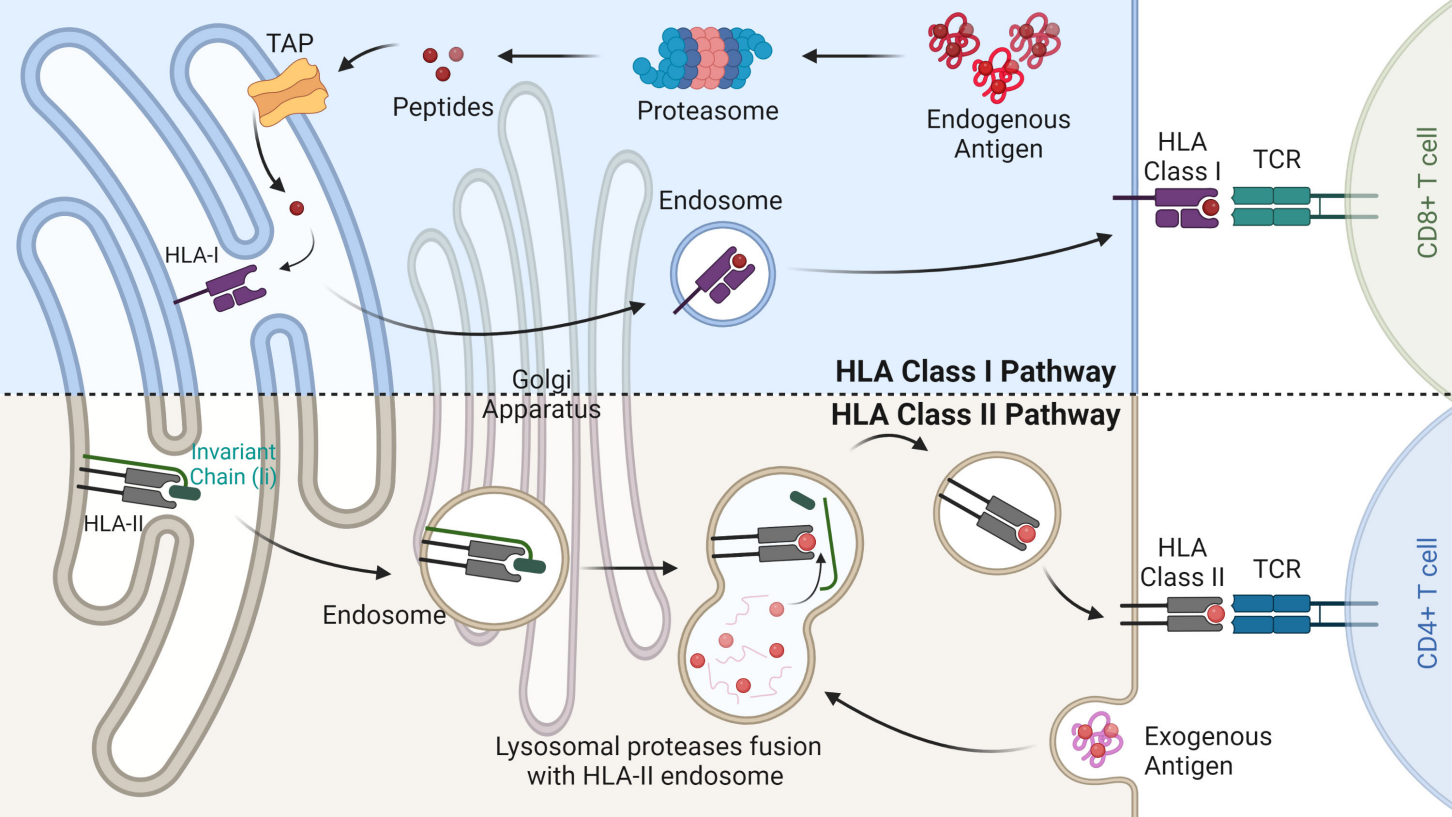
HLA class I and II antigen-presentation pathways. The HLA class I pathway (above) is responsible for degrading endogenous antigens into peptides via the multi-catalytic proteasome complex. Peptides are then transported into the endoplasmic reticulum (ER) by the transporter associated with antigen processing (TAP). Here peptides bind based on their relative affinity to the allotypes present. HLA-I peptide complex is then transported through the Golgi apparatus to the cell surface where it is scrutinised by CD8+ T cells. The HLA class II pathway (below) involves the degradation of exogenous antigens in the endosome compartment. In the ER immature HLA-II proteins are stabilised by the invariant chain and released into the HLA class II compartment. Here, the enzyme HLA-DM removes the class II invariant chain peptide (CLIP) from the binding pocket allowing for antigenic peptide binding. Mature HLA-II molecules bound to their peptide cargo are then transported to the cell surface for CD4+ T cell recognition.

Since the discovery of the first human leukocyte antigen (HLA) restricted peptide, a plethora of studies have sought to elucidate the peptide antigens that are presented during the development of adaptive immunity (1). The series of peptides presented on the cell surface by HLA molecules to be surveilled by CD4 and CD8+ T cells, is collectively termed the immunopeptidome (2) (**Figure 1**). The in-depth interrogation of these HLA peptides offers unique insights into the interplay between cells (whether healthy or perturbed) and the adaptive immune system. Characterising and understanding the immunopeptidome provides researchers with an excellent opportunity to develop precision therapeutics against cancer. Immunopeptidome discovery and analysis is directly dependent on the advancement of mass spectrometry, which in recent years has substantially progressed the field. In particular, the increased sensitivity, resolution and speed of mass spectrometers have allowed for tremendous leaps in the number of identifications of HLA-bound peptides in complex samples. This has facilitated the discovery of over 80,000 peptides in a single cell line, highlighting the substantial depth possible in comparison to 10 years ago, where only a handful of peptides were identified (3–6).

To this day, there is still an urgent need to circumvent the current drawbacks associated with immunopeptidomic analysis, these include the quantity of input material needed and the laborious nature required to isolate and analyse HLA bound peptides which become a major bottleneck when we want to analyse a big cohort of clinical samples (7–9). The majority of protocols currently in use either require manual processing for each sample or are designed for high throughput studies that necessitate the use of several 96-well plates to complete the entire process (7, 10–14)

We have developed a semi-automated immunopeptidomics method with mid-throughput capabilities, suitable for mid-sized cohorts and comparative immunopeptidomics studies. This protocol utilises a single 96-well plate and can process up to 12 samples per run, covering all the steps from loading anti-HLA antibody cocktails on magnetic microparticles to eluting purified HLA class complexes.

To evaluate the effectiveness of this protocol, we processed 12 samples in parallel (at cell counts of 5e5, 5e6, 1e7, and 5e7) with each sample processed in three biological replicates. We incorporated hyper-porous magnetic protein A beads to improve reproducibility, a KingFisherDuo liquid handling machine to reduce the potential for human error and decrease sample preparation time, and a data-independent acquisition (DIA) based approach to quantify HLA-bound peptides with high confidence and improve sensitivity and reproducibility.

## 2 Protocol

### 2.1 Reagents

- CHAPS Detergent (3-((3-cholamidopropyl) dimethylammonio)-1-propanesulfonate) (Thermo Scientific #28300)
- Phosphate buffered saline (PBS) (Sigma #P5493)
- Tris buffered saline (TBS) (Sigma #T5912)
- W6/32 Antibody (Leinco Technologies #H263)
- Acetonitrile (Thermo Scientific #FSBA955)
- Protease and Phosphatase inhibitor single use (Thermo Scientific #78442)
- iRT peptides (Biognosys - 11 iRT peptides)
- Sodium Chloride (Sigma #S9625)
- Trifluoroacetic acid (TFA) (Thermo Scientific #FSBA116)
- Tris (Sigma #10812846001)

### 2.2 Additional materials/equipment

- Tissuelyser LT (Qiagen #85600)
- Kingfisher Duo (Thermo Scientific #5400110)
- C18 stage tips (Thermo Scientific #87784)
- KingFisher tip comb (Thermo Scientific #97003500)
- KingFisher 96 well plate (Thermo Scientific #95040450)
- MagReSyn^®^ Protein A Max (Resyn Biosciences)
- Eppendorf Lobind 96 500 µL well deep plate (Eppendorf #30504305)
- Eppendorf Lobind 1.5 mL microcentrifuge tubes (Eppendorf #30108116)
- DynaMag-2 (Thermo Scientific #12321D)
- 1.0 mm Zirconium beads (Sigma #BMSD113210TP)
- 5 mm Stainless Steel beads (Qiagen #69989)
- epT.I.P.S.^®^ 2 – 200 µL (Eppendorf #30073436)
- epT.I.P.S.^®^ 50 – 1,000 µL (Eppendorf #30073436)

## 3 Procedure

### 3.1 Cell culture

MDA-MB-231 (triple negative breast cancer) cells were cultured in DMEM supplemented with 10% fetal bovine serum, 1% Penicillin/streptomycin and L-glutamine (2 mM) (Gibco) at 37°C with 5% CO2. Cells were treated with 50 IU of lyophilised human IFNγ (Miltenyi Biotec #130-096-484) for 48 h as per *Goncalves et al. (2021).* Cells were grown to 5e5, 5e6, 1e7 and 5e7 in three biological replicates and centrifuged at 2700 g, snap frozen with liquid nitrogen and stored at -80°C.

### 3.2 Tissue and cell lysis homogenisation (~1.5 h)

**Prepare Lysis Buffer:**

Technical Note: Keep lysis buffer and PBS on ice (0- 4˚C).

- 1% (w/v) CHAPS
- 50mM Tris pH 8
- 150 mM NaCl
- Half Protease and Phosphatase inhibitor single use cocktail (100X)

For homogenisation of cell pellets, use 0.5mm zirconia beads.

Technical Note: For tissue samples, use the 5mm stainless steel beads (one bead per sample). For tougher tissue, 7 mm beads can be used to improve disruption efficiency. The method described below is for the TissueLyser LT but can be adapted to other homogeniser systems.

**Figure 2:**
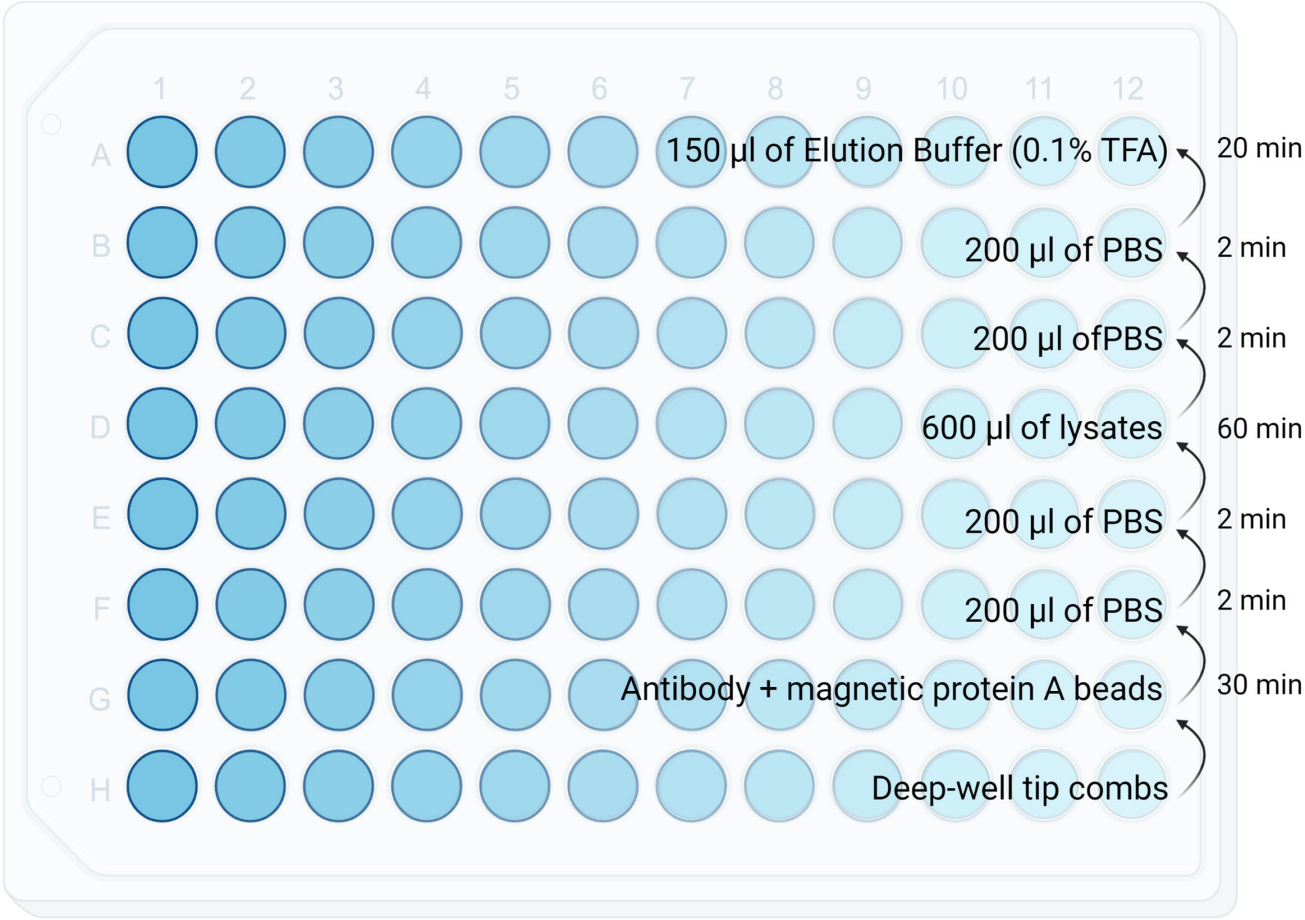
Diagram showing reagents plating format for the KingFisher. Volumes as well as the KingFisher method timing is specified in each row.

1. Prepare lysis buffer on ice.
2. Transfer frozen samples onto ice and immediately add 300 - 600 µL of lysis buffer as well as the homogenisation beads to each tubes.
3. Transfer the tubes into the **TissueLyser LT** and homogenise for 2-5 min at 50 Hz. The duration of disruption and homogenisation depends on the tissue being processed and can be extended until no tissue debris is visible.
4. Mix gently and then leave rolling at 4˚C for 1 hour.
5. Transfer the lysate to a clean LoBind eppendorf microcentrifuge tube. Wash the homogenisation beads with 200 ul of fresh lysis buffer and collect it in the same sample tube.
6. Spin lysates at 18000g for 10 min at 4˚C.
7. Transfer the supernatant to a KingFisher 96 well plate (**Figure 2**).

### 3.3 Preparing magnetic beads bound to the Antibody (~10min)

**MagReSyn preparation:**

1. Resuspend MagReSyn^®^ Protein A MAX thoroughly by vortex mixing or inversion to ensure a homogenous suspension (**Table 1**).
2. Immediately transfer MagReSyn^®^ Protein A MAX.
3. Technical Note: Use LoBind pipette tips. Pipette slowly as the storage buffer will tend to stick to the side if pipetting too fast.
4. Place the tube on the magnetic separator and allow the microparticles to clear for 30sec. Remove the storage buffer without disturbing the microparticles.
5. Wash the microparticles in 2X volume of binding buffer (i.e. PBS/TBS). Allow a minimum of 30 sec for microparticle equilibration.
6. Place the tube on the magnetic separator and allow the microparticles to clear for 30sec. Remove the binding buffer by aspiration with a pipette and discard.
7. Repeat steps 4 and 5 twice (total of 3 washes).
8. MagReSyn^®^ Protein A MAX is ready for antibody binding. Resuspend washed beads in 200 ul of PBS/TBS for each sample (**Table 1**). Note: MagReSyn Protein A Max has an antibody capacity of 320ug Rabbit IgG/mg beads
9. Transfer an equivalent amount of microparticles/antibody solutions into the KingFisher 96 well plate (**Figure 2**).

**Table 1:** Ratio of magnetic beads and antibody.

Number of cells	MagReSyn (ul)	MagReSyn (ug)	W6/32 (Anti HLA-I) ug
5e7 or less	80 ul	1250ug	500ug

### 3.4 KingFisher plate preparation and immunoaffinity purification (~2 h)

1. Setup the KingFisher plate according to **Figure 2**

2. KingFisher steps is summarised in **Figure 2** (Method file can be made available on request for all KingFisher models)

Technical Note: The throughput can be further scaled up to 96 samples in parallel, processed in approximately 120 min, by utilising a KingFisher™ Flex or Apex magnetic bead handling stations, without the need for additional method re-optimization.

### 3.5 C18 clean-up (~1.5 h)

**Figure 3:**
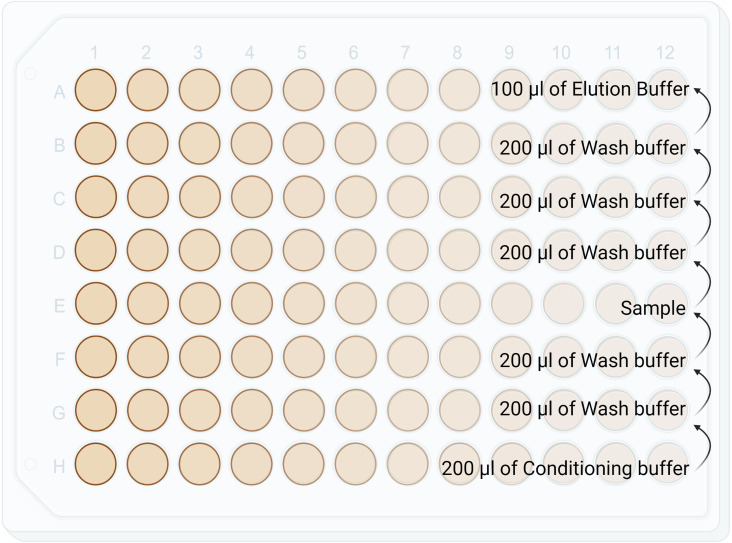
Diagram showing reagents plating format for the C18 stage tip clean up in a Lobind 96 well plate. This step is manual and not performed by KingFisher Duo. Conditioning buffer: 50%ACN/0.1% TFA. Wash buffer: 0.1% TFA. HLA-I Elution Buffer: 28% ACN/ 0.1% TFA. HLA-II Elution Buffer: 32% ACN/ 0.1% TFA.

Technical Note:

- This step can be performed on a separate day. If it is the following day, keep samples in 4˚C, otherwise in -80˚C.
- If your C18 column capacity is less than 300ug, it is advisable to repeat the clean-up twice to maximise recovery of the peptides. This is due to C18 also binding to eluted HLA class I and antibody molecules.

1. Set up the 96 well plate as shown in **Figure 3**.

2. Condition C18 stage tips by pipetting up and down 2-3 times in conditioning buffer (50% ACN/0.1% TFA)

3. Wash column by pipetting up and down 2-3 times in wash buffer (0.1% TFA)

4. Collect sample in C18 column by pipetting up and down 20 times

5. Wash sample and C18 column 2-3 times in wash buffer.

6. Elute the peptides from C18 column by pipetting up and down 20 times in recommended elution buffers (28% ACN/0.1% TFA for HLA-I and 32% ACN/0.1% TFA for HLA-II).

7. Dry sample using a vacuum evaporator.

8. Prior to mass spectrometry analysis, resuspend sample in 12ul of 2% ACN/0.1% TFA

9. Sonicate 10 min in water bath sonicator

10. Centrifuge at 18000g for 20 min

11. Transfer to a MS vial

### 3.6 Mass spectrometry analysis

All samples were analysed on a Exploris 480 orbitrap mass spectrometer (ThermoFisher Scientific) coupled online to a RSLC nano HPLC (Ultimate 3000 UHPLC, ThermoFisher Scientific). The mass spectrometer was operated in DIA mode. Each sample was resuspended in 12ul of loading buffer with 6ul injected onto a 100 μm, 2 cm nanoviper Pepmap100 trap column, eluted and separation performed on a RSLC nano column 75 μm x 50 cm, Pepmap100 C18 analytical column (ThermoFisher Scientific). The separation was performed at a flow rate of 250 nl/min by a gradient of 0.1% formic acid in water (solvent A) and 80% acetonitrile/0.1% formic acid (solvent B).

The eluent was nebulised and ionised using a nano electrospray source (ThermoFisher Scientific) with a distal coated fused silica emitter (Trajan). The capillary voltage was set at 1.9 kV. MS1 scan range from 370 to 1,675 m/z with a resolution of 120,000 (at m/z 200) using a custom AGC target of 200%, a maximum ion injection time set to auto and 22 variable window DIA MS/MS scans in the orbitrap. The variable windows were calculated using Sciex excel calculator (**Supplementary Table 1**) (15); Each MS2 scan was acquired within a scan range of 120 - 1,450 m/z at a resolution of 30,000 (at m/z 200) using a custom AGC target of 1000% with HCD collision energy of 27% and the overlap between consecutive MS/MS scans was set to 1 m/z.

### 3.7 Data analysis

Spectral libraries were generated using the Pulsar engine in Spectronaut (version 16.2 - Biognosys) with the following settings: (i) digest set to no enzyme and unspecific mode and (ii) Oxidation (M) was set as a variable modification. The DIA data was searched using the settings as described in the **Supplementary Materials**. No imputations or normalisations were performed across samples during data analysis. Missing values were marked as “Filtered” in the **Supplementary Data 1**.

### 3.8 Binding prediction of HLA peptides

Peptides were allocated as binders or non-binders using NetMHCpan4.1 (16). This software predicts HLA-peptide binding using artificial neural networks, here we implemented the default cut-off of a rank score of <2 as a binder peptide.

## 4 Results

**Figure 4:**
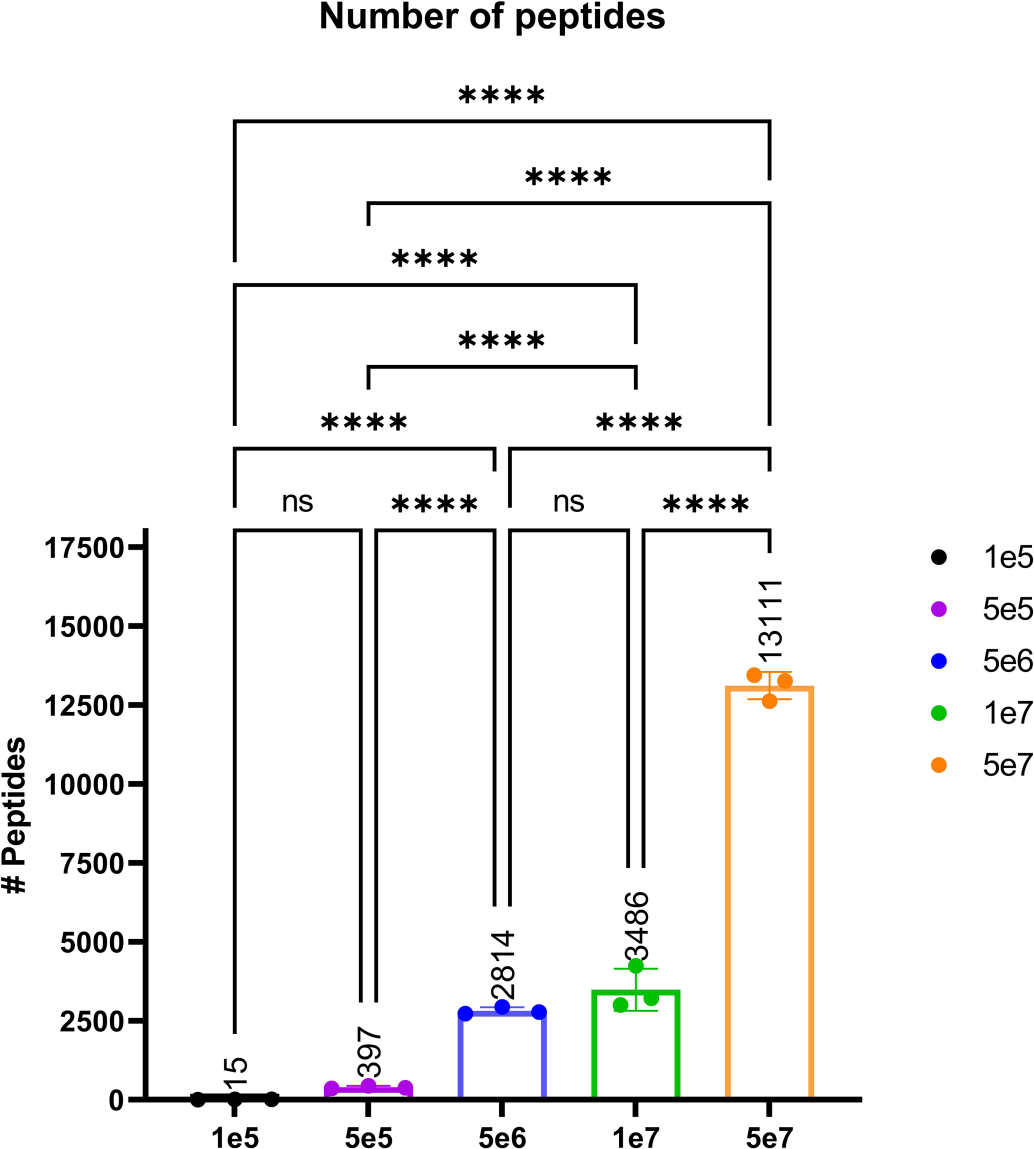
The average number of peptides per cell count pellet. The number of peptides identified for each sample were as follows: 1e5 (black), 5e5 (purple), 5e6 (blue), 1e7 (green), and 5e7 (orange). Please note that the experiment for the 1e5 samples was performed separately to evaluate the limit of detection. Statistical significance with a p-value < 0.0001, as determined by an one-way ANOVAa t-test, is denoted by ****.

**Figure 5:**
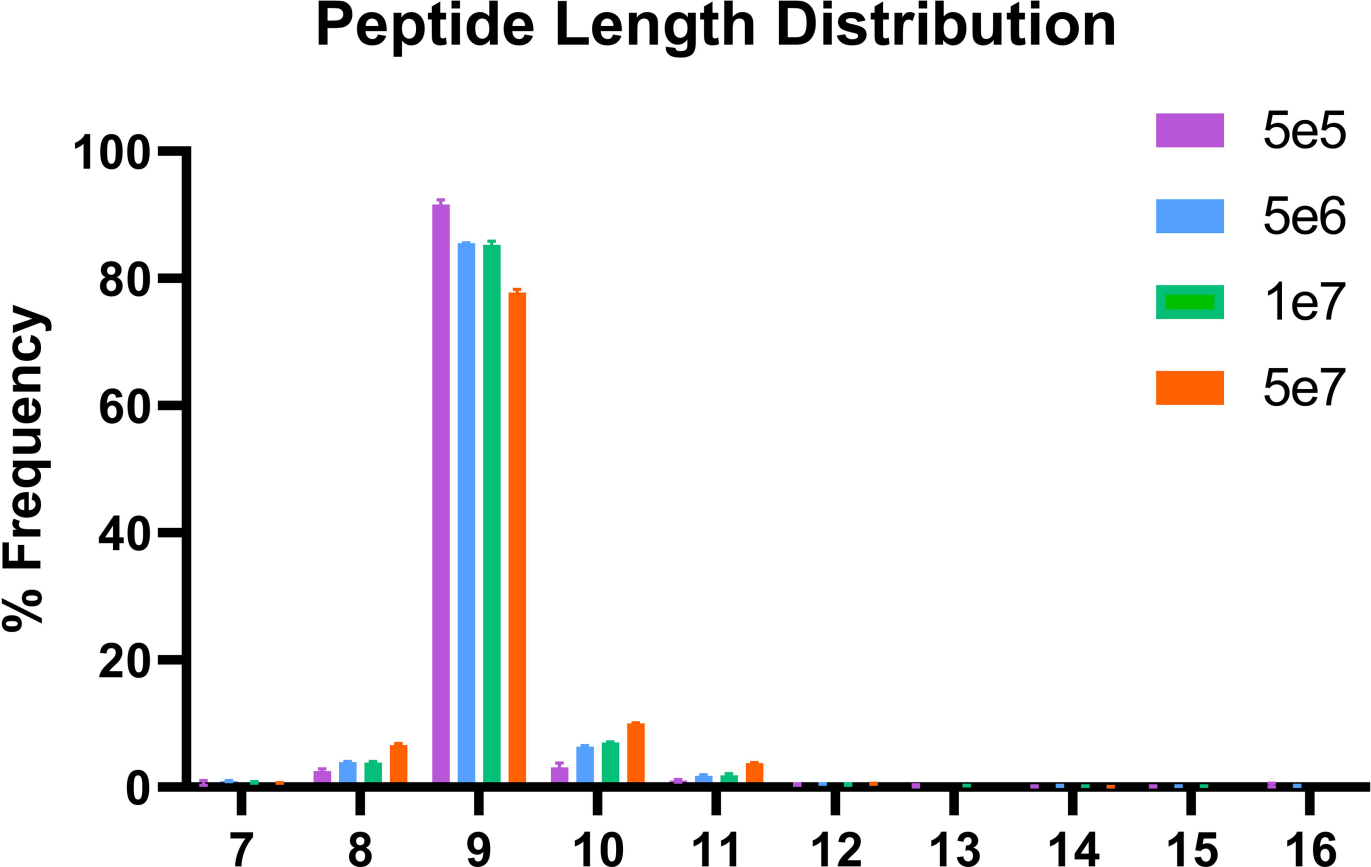
Length distribution of peptides bound to HLA class I. The x-axis shows the length of the peptides for each condition and the y-axis the percentage frequency for each length.

**Figure 6:**
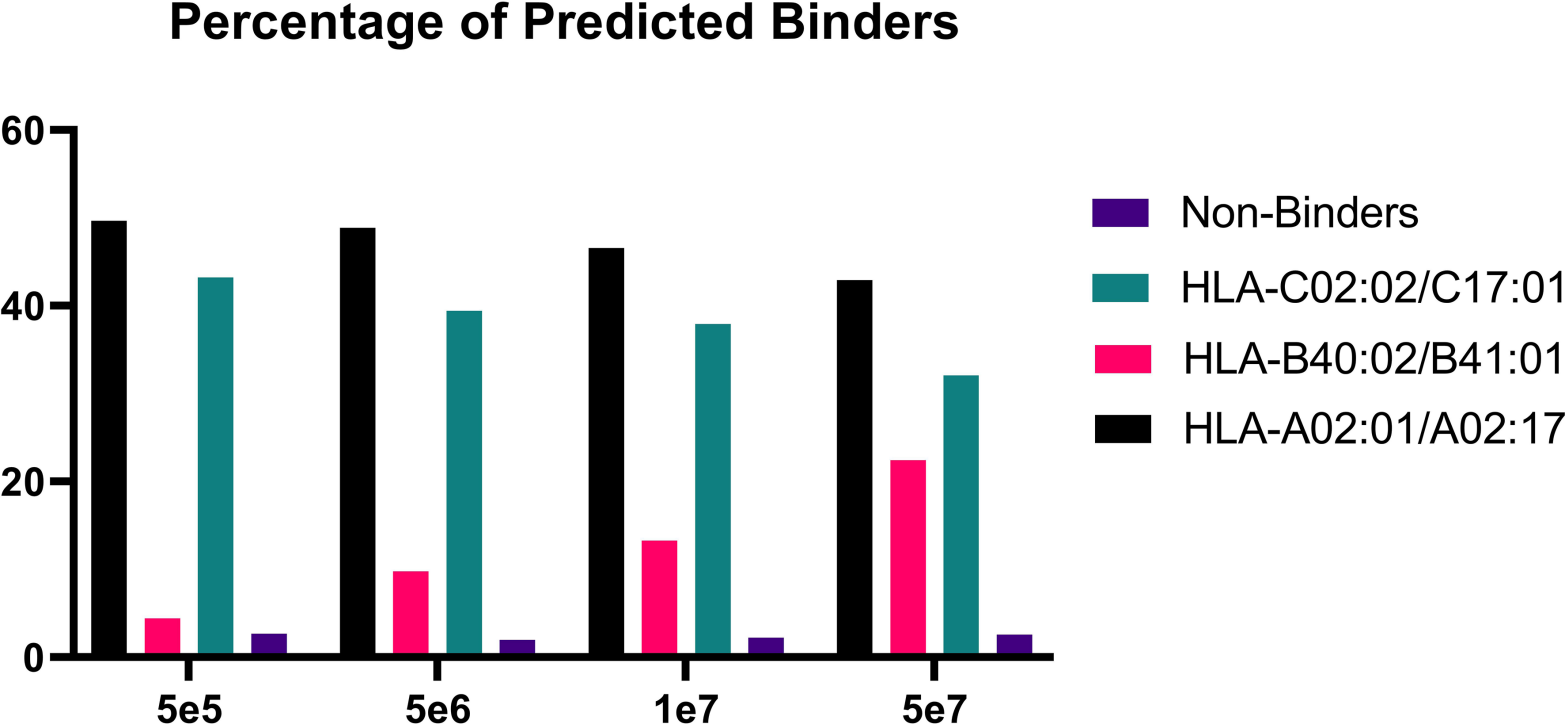
Percentage of predicted binders versus non-binders. Bar graph showing the percentage of binders for each condition following binding predictions on NetMHCpan4.1 (16).

In order to validate our method, we profiled the peptides liberated from HLA molecules on the MDA-MB-231 cell line to determine their characteristics. Using this semi-automated approach, we identify 13,312 unique HLA-I peptides across all samples at 1% FDR. When we compare the average number of peptides across each replicate, we observe 13,111 HLA bound peptides from eluates isolated from 5e7 cell pellet samples, 3,486 peptides from 1e7 samples, 2,814 from 5e6 and 397 from 5e5 cell pellets (**Figure 4**). In a separate experiment, we studied the immunopeptidome of three 1e5 cell pellets to determine our limit of detection with only a handful of peptides identified in each sample (**Figure 4**). As expected for HLA class I, all samples contain a high proportion of 9mers in comparison to other peptide lengths (**Figure 5**). In order to ascertain whether these HLA peptides are indicative of the allotypes expressed on the MDA-MB-231 cell line, we examined the predicted binding of peptides between 8 and 13 amino acids in length to the HLA alleles expressed on this cell line. This showed ~96% of peptides were binders using NetMHCpan (16) (**Figure 6**).

**Figure 7:**
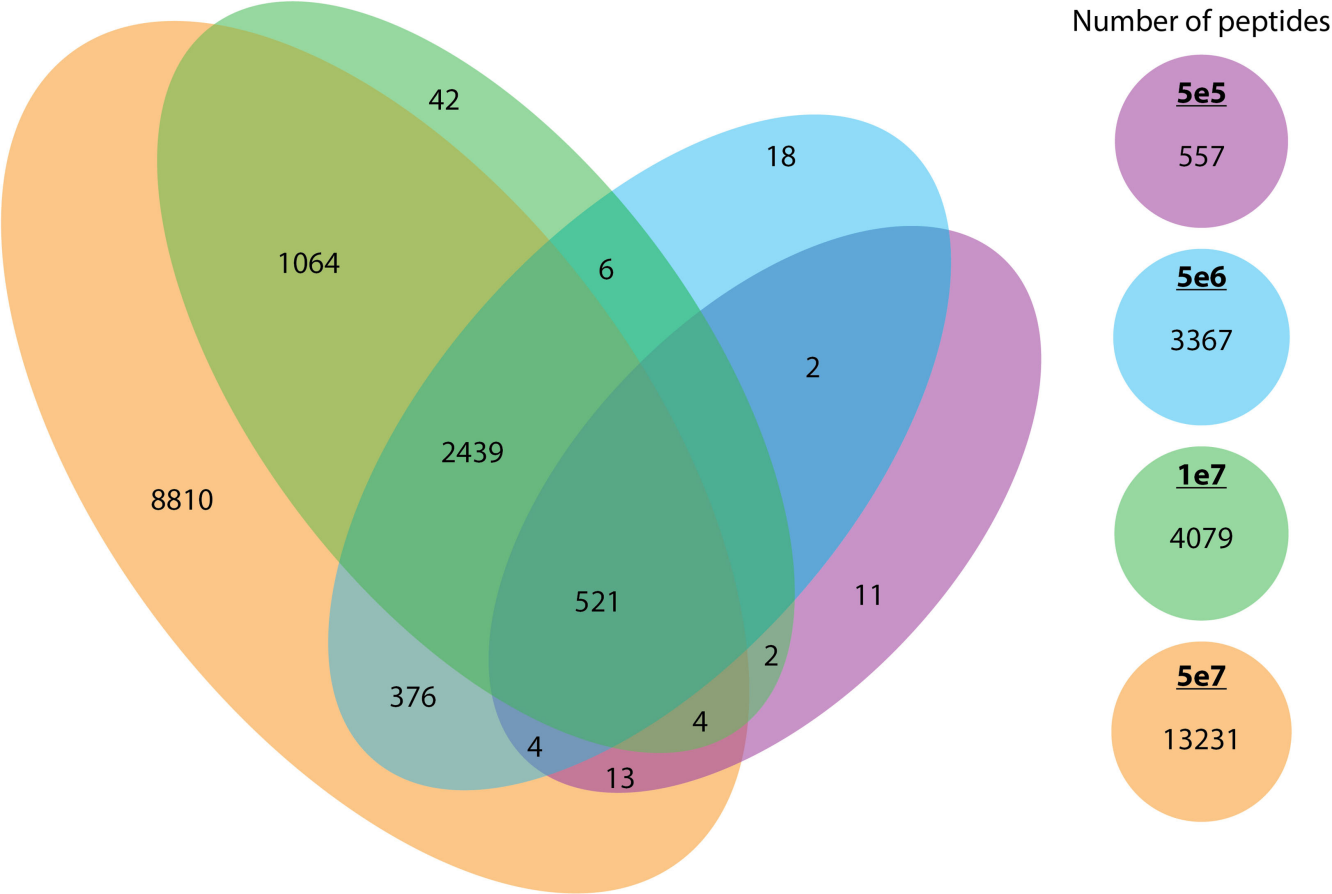
The overlap between peptides identified across different cell counts. The overlap of unique peptides across different cell counts (5e5 in purple, 5e6 in blue, 1e7 in green, and 5e7 in orange) is represented in a Venn diagram.

As anticipated, increasing the cell number resulted in an expansion of the peptide repertoire. We compared the peptides identified across each cell count and observed that the majority of peptides identified in smaller pellets were also identified in larger pellets. We observed that the 5e7 cell samples encompassed a very high proportion of the identified peptides in smaller pellets with less than 1% of the peptides been missed. (**Figure 7**).

**Figure 8:**
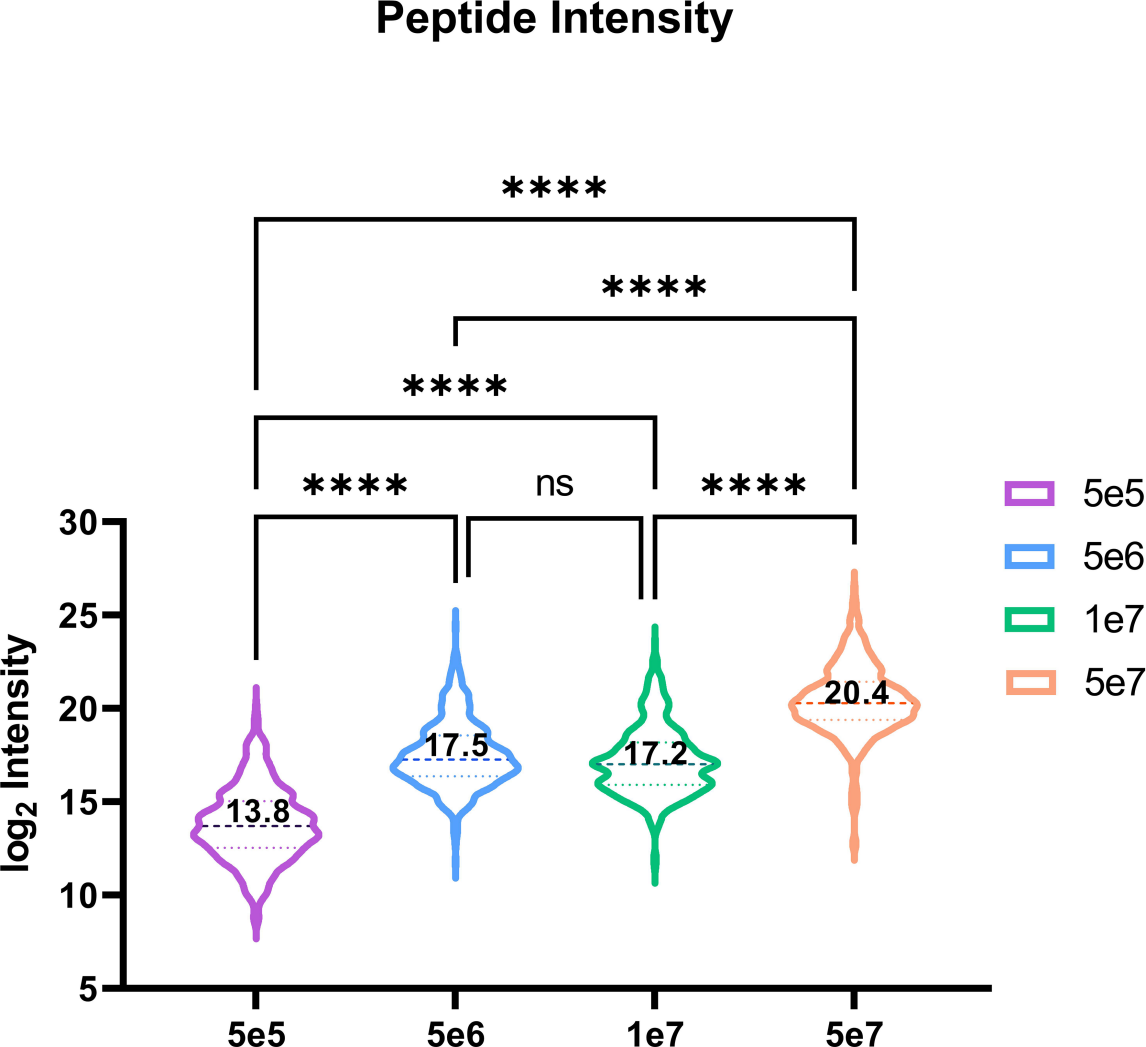
Violin plot depicting the peptide intensity between commonly identified HLA peptides. Violin plot showing the trend in intensities for the 521 overlapping peptides across four conditions. The mean log2 intensities for each condition are indicated by the values. Statistical significance with a p-value < 0.0001, as determined by an one-way ANOVA, is denoted by ****.

Utilising our optimised DIA variable window approach, we compared the relative intensities of commonly identified peptides in all four conditions predicted (n=521). Our observations indicate an increasing trend corresponding to log2 mean intensities, with a ~13-fold difference between 5e5 and 5e6, a ~97-fold difference between 5e5 and 5e7, and a ~7-fold change between 5e6 and 5e7. However, we did not observe a significant difference in the intensity of common ions between 5e6 and 1e7 pellets (**Figure 8**).

## 5 Discussion

The identification of HLA-bound peptides is crucial in the development of T cell-based immunotherapy, where potential epitopes are recognised by the adaptive immune system and used to design peptide-based vaccines for targeted elimination of diseased cells. Interest in this field has increased exponentially, leading to the development of novel protocols, the expansion of search algorithms that are not limited to reference proteomes and the development of different initiatives that have driven data sharing and bolstered data repositories (17–26). However, a constant “Achilles Heel’’ in this space has always been the difficult and laborious nature of immunopeptidomic workflows, with large sample material needed, high resolution mass spectrometers required and the lack of standardised protocols to ascertain HLA peptides. These limitations have collectively hindered the growth of this field and served as a constant challenge to overcome (17, 18, 25, 26).

Here, we have developed and showcased SAPrIm, A Semi-Automated Protocol for Mid-Throughput Immunopeptidomics and address some of the current limitations in this space.

Using MDA-MB-231 cell line as a model, we have demonstrated that even with limited starting material (5e5 cells), we can concordantly identify and quantify HLA bound peptides peptides that recapitulate the expected HLA peptide characteristics observed at both 10 and 100 times the starting material. Our results demonstrate that significant depth of coverage can be achieved using this protocol, as evidenced by the identification of approximately 13,000 peptides concordantly from 5e7 cells. Notably, this was accomplished without the need for offline fractionation methods and with a sample preparation time of less than 4 hours, with around 2 hours of the preparation time being hands-off experiments. We highlight that SAPrIm not only reduces manual handling time but exceeds current identification rates observed with other protocols (7, 11, 12).

When we perform comparative analysis, we see a high degree of overlap between each input amount highlighting the reproducibility of our approach. Furthermore, using this workflow we identify 697 peptides derived from known 195 cancer antigens highlighting that even with limited input material we are able to identify actionable targets for T cell mediated immunotherapy. Of note, in 5e5 cells we identify 36 peptides derived from 28 cancer antigens (**Supplementary Data 1**).

Although DIA was initially introduced as a quantitative method in proteomics, recent advancements in data acquisition and analysis have made it a suitable method for both discovery and quantitative immunopeptidomics (14). This is due to the development of techniques such as spectral library-free searches, the generation of pan-spectral libraries from publicly available data, and the use of MS2 prediction algorithms for generating spectral libraries (14, 27). These improvements have expanded the capabilities of DIA, making it a valuable tool for researchers in the field of immunopeptidomics.

Although not performed in this study, it is possible to label the 12 samples resulting from the SAPrIm protocol with TMT tags using more than 12 channels and analyse them in a single LC-MS/MS run (28, 29). Additionally, the SAPriM protocol has the potential to be used for analysing HLA-II peptides and could be scaled up to analyse up to 96 samples using the KingFisher Apex system (Thermo Fisher).

Taken together, the use of this semi-automated approach facilitates many of the current limitations when it comes to the immunopeptidomic space. SAPrIm automates this approach, cuts sample preparation time and reduces the complexity of sample preparation. In addition to this, we support the feasibility of using relatively low quantities of antibody and starting material in comparison to traditional workflows, without the need for crosslinking (5, 7). We have also integrated this with the use of DIA, allowing for the accurate quantification and mapping of all the ions/peptides for future re-inspections of the data. A reliable and efficient workflow with a short turnover time is crucial in research and clinical settings to confidently screen biological and clinical materials for tumour-specific epitopes. These capabilities make SAPrIm technology a promising approach for translational immunopeptidomics.

## 6 Troubleshooting

### 6.1 Samples are highly viscous after lysis (Step 1.1)

This is caused by the release of genomic DNA and is completely normal. This could be due to higher concentration of detergent. Consider decreasing the detergent concentration and/or increase the homogenisation timing.

### 6.2 Cloudy top layer after centrifugation (Step 1.6)

Depending on the sample type, the fatty material can aggregate as a thin top cloudy layer post centrifugation. In our experience, this did not interfere with the immunoaffinity purification.

### 6.3 Uneven distribution of magnetic beads between samples (Step 3.8)

The beads settle very quickly, and care must be taken to ensure that equal volumes of beads are dispensed into each sample. Consider vortexing the beads, or mixing by inversion, immediately before pipetting each (and every) sample.

### 6.4 Low peptide yield (Step 5.11)

If the yield is lower than expected following data analysis, several parameters in the workflow should be checked, including cell lysis, antibody stability/batch, HLA expression (where possible) as well as the mass spectrometer performance (e.g using Hela digest or Glu-1-Fibrinopeptide B peptide standard). Different tissue types would require different amounts of time and/or type of beads for homogenisation. Buffer’s pH for antibody/beads coupling as well as immunoaffinity purification steps are critical and should be kept at ~pH 8.

## Data availability statement

The datasets presented in this study can be found in online repositories. The names of the repository/repositories and accession number(s) can be found below: ProteomeXchange Consortium via the PRIDE (30) partner repository with the dataset identifier PXD041046.

## Author contributions

PF, TLKS, SS and GG conceived and designed the method. GG, TLKS, TS, JS, LB, DJ, MS and TLKS performed experiments and collected data. GG and TLKS performed the data analysis. GG, AWP, SR, PF and TLKS wrote the manuscript. All authors contributed to the article and approved the submitted version.

## Supplementary material

Supplementary Figure 1 SDS-PAGE gel stained with coomassie showing the bind of antibody to the Magresyn protein A with and without crosslinking with dimethyl pimelimidate (DMP).

Supplementary Figure 2 Venn Diagram showing the overlap between the replicates in each condition

Supplementary Table 1 Variable DIA acquisition windows for immunopeptidomics analysis

Supplementary Data Sheet 1 Excel sheets summarising the presence of peptides in each replicate.

Supplementary Materials Information about the Spectronaut search settings.
