# Supplementary figures and images for: SAPrIm, a semi-automated protocol for mid-throughput immunopeptidomics
Source: Front Immunol. 2023 Jun 2;14:1107576. doi: 10.3389/fimmu.2023.1107576 (PMC10272402; doi:10.3389/fimmu.2023.1107576)

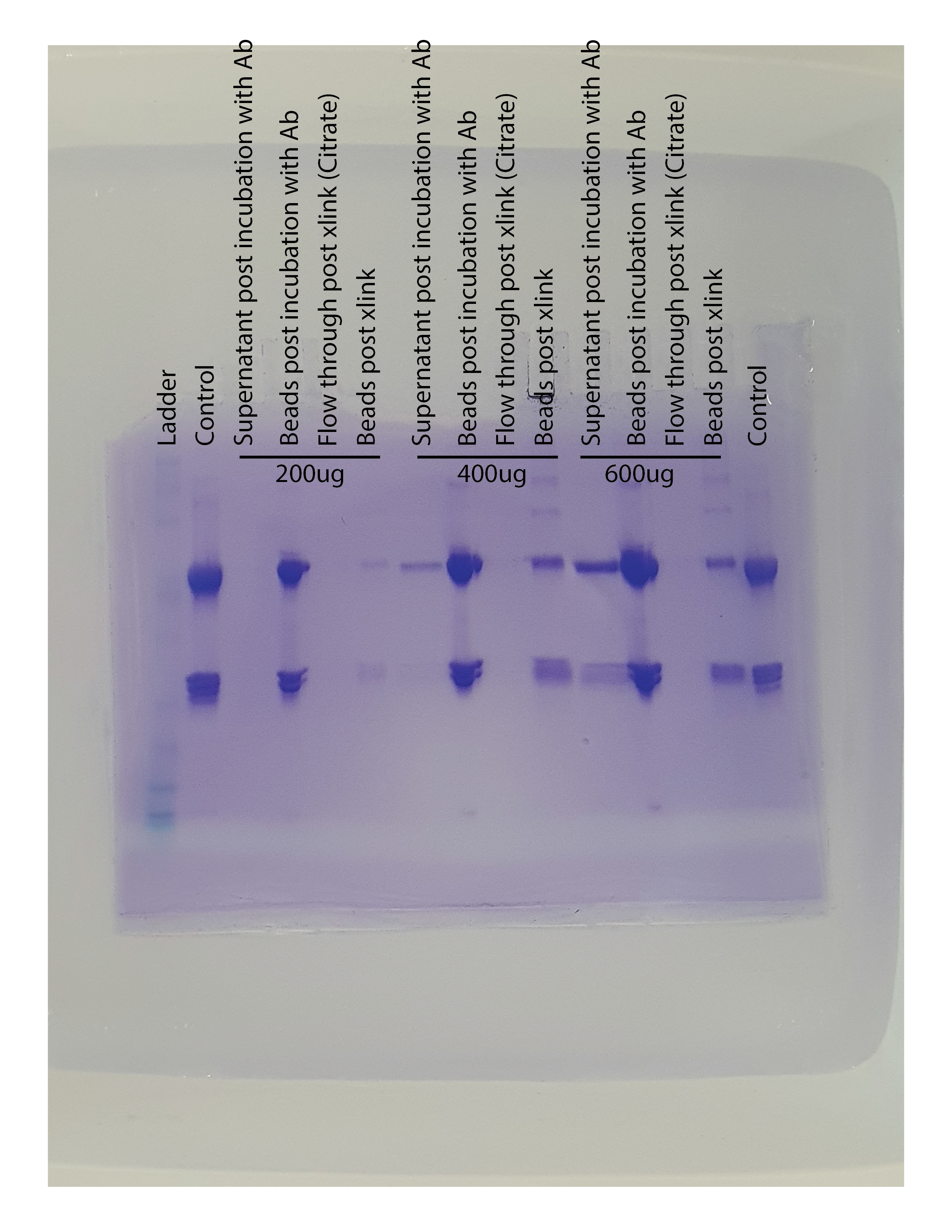

Supplement: Supplementary Figure 1 — SDS-PAGE gel stained with coomassie showing the bind of antibody to the Magresyn protein A with and without crosslinking with dimethyl pimelimidate (DMP). [file Image_1.jpg]

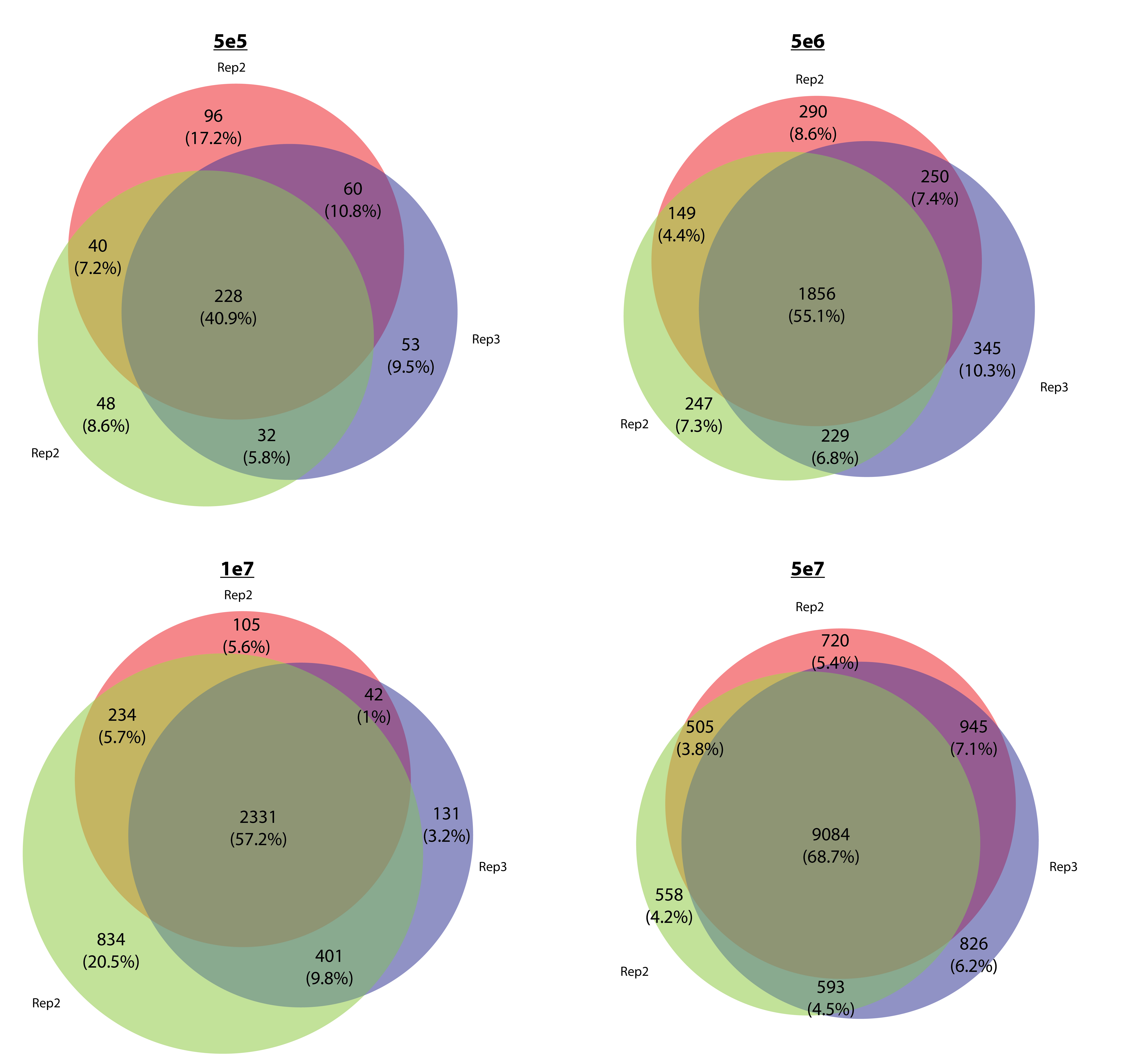

Supplement: Supplementary Figure 2 — Venn Diagram showing the overlap between the replicates in each condition [file Image_2.jpg]
